# Interaction between Hepatitis B Virus and Toll-Like Receptors: Current Status and Potential Therapeutic Use for Chronic Hepatitis B

**DOI:** 10.3390/vaccines6010006

**Published:** 2018-01-16

**Authors:** Zhiyong Ma, Qian Cao, Yong Xiong, Ejuan Zhang, Mengji Lu

**Affiliations:** 1 Department of Infectious Diseases, Zhongnan Hospital of Wuhan University, Wuhan 430071, China; mzy2001pl@163.com (Z.M.); qiancao2015@126.com (Q.C.); yongxiong64@163.com (Y.X.); 2 Wuhan Institute of Virology, Chinese Academy of Sciences, Wuhan 430071, China; zhangejuan@wh.iov.cn; 3 Institute of Virology, University Hospital Essen, University of Duisburg-Essen, 45122 Essen, Germany

**Keywords:** hepatitis B virus, toll-like receptors, innate immunity, adaptive immunity, immunotherapy

## Abstract

Immune defense against infection with the hepatitis B virus (HBV) is complex and involves both host innate and adaptive immune systems. It is well accepted that the development of sufficient HBV-specific T cell and B cell responses are required for controlling an HBV infection. However, the contribution of innate immunity to removing HBV has been explored in recent years. Toll-like receptors (TLRs) are recognized as the first line of antiviral immunity because they initiate intracellular signaling pathways to induce antiviral mediators such as interferons (IFNs) and other cytokines. Recent studies show that the activation of TLR-mediated signaling pathways results in a suppression of HBV replication in vitro and in vivo. However, HBV has also evolved strategies to counter TLR responses including the suppression of TLR expression and the blockage of downstream signaling pathways. Antiviral treatment in chronic HBV-infected patients leads to an upregulation of TLR expression and the restoration of its innate antiviral functions. Thus, TLR activation may serve as an additional immunotherapeutic option for treating chronic HBV infection in combination with antiviral treatment.

## 1. Introduction

Immune defense against hepatitis B virus (HBV) infection is complex and involves both host innate and adaptive immune systems [1,2]. During an acute HBV infection, robust polyclones and multi-specific CD8^+^ T cell and CD4^+^ T cell responses against HBV proteins are observed. This is due to early phase clearance of the virus by both noncytolytic and cytolytic mechanisms [3,4,5]. Nonetheless, patients with chronic HBV infection are usually associated with exhaustion of HBV-specific CD8^+^ T cell and CD4^+^ T cell responses. The exhaustion phenotype is characterized by poor cytotoxic activity, impaired cytokine production, and high expression of multiple inhibitor molecules such as programmed cell death-1 (PD-1), the lymphocyte activation gene-3 (LAG-3), and the cytotoxic T lymphocyte-associated antigen-4 (CTLA-4) [6,7]. The key role of HBV-specific immune responses in terminating HBV infection is well established. However, the contribution of innate immunity in this regard has only been explored in recent years.

During the course of infection, microbe pathogens are initially recognized by the innate immune system due to the way pathogen-associated molecular patterns (PAMPs) interact with host pattern recognition receptors (PPRs). PPRs include toll-like receptors (TLRs), RIG-I-like receptors (RLRs), NOD-like receptors (NLRs), and C-type lectin receptors (CLRs) as well as diversified DNA receptors [8,9]. HBV is called a “stealth virus” that induces negligible interferons (IFNs) or IFN-stimulated genes (ISGs) in acute HBV-infected chimpanzees [10] and patients [11,12]. However, recent studies reveal that HBV infection can be recognized by host PPRs, which then induce a cytokine response that restricts the replication of the virus in newly developed experimental systems [13,14,15]. Importantly, several institutions have found that TLRs mediate the inhibition of HBV replication in hepatocytes and animal models [16,17,18,19,20]. However, during chronic HBV infection, the expression of TLRs on hepatocytes, hepatic cells, and peripheral immune cells was changed while TLR functions were impaired [21,22,23,24]. A recent study also shows that TLR-mediated signaling pathways are essential for eliciting functional HBV-specific CD8^+^ T cell responses in vivo [25]. Those studies demonstrate the complicated interaction between HBV and TLRs. The understanding of this process is important for the development of TLR agonists as immune-therapeutics for chronic HBV infection.

## 2. The Interaction between HBV and TLRs

### 2.1. Recognition of HBV by Innate Immunity

The recognition of HBV by innate immunity may involve three types of host cells: (1) the hepatocytes; (2) hepatic non-parenchymal cells (NPCs) such as liver sinusoidal endothelial cells (LSECs) and Kupffer cells (KCs); and (3) innate immune cells like myeloid or plasmacytoid dendritic cells (DCs) and macrophages.

A recent study demonstrated that primary human hepatocytes (PHHs) as well as differentiated HepaRG (dHepaRG) cells express functionally at relevant levels of retinoic acid-inducible gene-1 (RIG-I), melanoma differentiation-associated protein 5 (MDA5), and most of the TLRs except for TLR7 and TLR9 [26]. Baculoviral vector-mediated or physiologic HBV infection of dHepaRG cells led to the production of pro-inflammatory cytokines such as IL-6 and type I IFNs, which suggests that hepatocytes themselves could detect HBV and generate innate immune responses [14]. In another study, HBV infection of the micro-patterning and co-culturing of PHHs with fibroblasts (MPCC) with human infectious serum also led to the induction of type I and type III IFN responses. Notably, the IFN responses followed by HBV infection presented two peaks: a weak, early response peaked between 12 h to 48 h post infection and a stronger one appeared later between day seven and day 14 post-infection [15]. A recent study also demonstrated that a type III but not a type I IFN response could be induced via HBV replication in HepG2 cells and PHHs. In this cell culture model, the peak of the IFN response appeared between 24 h to 48 h post-infection [13]. Additionally, in a chimeric mice model, where more than 70% of murine hepatocytes were replaced by human hepatocytes, a similar hepatic type III IFN response could also be detected four or five weeks after HBV infection. The RIG-I mediated sensing of the 5′-ε structure of HBV pregenomic RNA was attributed to the induction of hepatic type III IFN response [13]. However, the opposite results were observed in a more recent study that used various human cell-culture models and humanized liver chimeric mice. The study shows an absence of the IFN responses despite robust HBV replication [27]. In contrast, the hepatitis C virus (HCV) infection activated the IFN responses both in vitro and in vivo. It seems that HBV behaves as a “stealth” virus and is not sensed by the intrinsic IFN-related innate immunity of infected hepatocytes, though macrophages respond to HBV exposure with cytokine production [27].

Hepatic NPCs including KCs and LSECs play an important role in initiating innate and adaptive immune responses against invasive hepatotropic pathogens [28]. Using cultured PHHs and NPCs, Hosel et al. showed that HBV was recognized by KCs. This recognition leads to nuclear factor kappa B (NF-κB) pathway activation and IL-6 production with no induction of type-I IFNs [29]. However, the mechanisms behind how KCs sense the virus are not clear and need further investigation. LSECs have been found to play a key role in the initial uptake of HBV and to then release the virus in order to infect adjacent hepatocytes [30]. A recent study demonstrated that LSECs could be activated by a highly virulent mouse hepatitis virus (type 3) through the TLR2-mediated signaling pathway. This led to severe onset of hepatitis associated with high levels of inflammatory mediators and the recruitment of inflammatory cells [31]. However, whether LSECs are involved in recognizing an HBV infection remains unknown.

HBV virions and components can be recognized by conventional innate immune cells such as DCs, monocytes, or macrophages once they enter the host. HBV nucleocapsid was initially considered a ligand of TLR2 because it could stimulate macrophages to produce proinflammatory cytokines in a TLR2-dependent manner in vitro [32]. However, in this study, the HBV nucleocapsid was derived from a bacterial expression system, which may be contaminated by TLR2 agonists like lipoproteins [33,34]. A recent study demonstrated that exposing myeloid DCs to HBsAg resulted in strong activation and maturation of the DCs, which enhanced its capacity to activate antigen-specific cytotoxic T cells. Further analysis suggested that CD14, a co-receptor of TLR4 in myeloid DCs, plays a crucial role in the HBsAg-mediated DCs maturation by interacting with and internalizing HBsAg into these cells [35].

To summarize, the host innate immunity could sense the HBV infection in vitro and in vivo. However, the underlying molecule mechanisms of the interaction of HBV and PRRs are poorly understood and further investigation into this field is urgently needed.

### 2.2. Expression and Function of TLRs are Impaired in Chronic HBV Infection

Several studies suggest that the expression and function of TLRs are impaired during chronic HBV infection. The reduction of TLR2 expression was discovered in hepatocytes and KCs among liver biopsies and peripheral blood mononuclear cells (PBMCs) isolated from patients with chronic hepatitis B (CHB). Interestingly, HBeAg was found to contribute to reducing TLR2 expression and impaired induction of TNF-α in hepatic cell lines [21]. After being tested with the TLR2 ligand, another study confirmed that the expression of TLR2 on PBMCs of CHB patients was reduced with an impaired cytokine production. This phenomenon was correlated with the plasma HBsAg levels but not HBeAg [22]. The reduced expression of TLR9 was also found in plasmacytoid DCs (pDCs) of CHB patients, which correlate with IFN-α secretion impairment after ex vivo stimulation with TLR9 ligands [23,36,37]. The decreased expression of TLR3 on PBMCs, hepatic KCs, and hepatocytes was also found in CHB patients when compared with health controls [24]. Interestingly, both the antiviral treatment with pegylated-interferon (PEG-IFN) and entecavir can restore the expression of TLR3. Additionally, PEG-IFN is more efficient than entecavir [24]. A recent study showed that an increased proportion of TLR2(+) monocytes was consistently associated with a better response to PEG-IFN-α in CHB patients under long term PEG-IFN-α treatment [38]. Another study also demonstrated that an elevated expression of TLR2 on monocytes was associated with HBeAg seroconversion as early as four weeks after initiation of Peg-IFN treatment. Once the seroconversion occurred, the TLR2 expression levels on monocytes grew significantly, which lead to TLR2-associated IL-6 production [39]. A recent study found that the expression and function of TLR8 on monocytes were impaired in CHB patients compared with health controls. As a result, they showed higher TLR8 expression at 12 weeks after PEG-IFN treatment could potentially predict the complete response rate of the patients [40]. These studies imply that antiviral treatment, especially PEG-IFN, could restore the expression and function of TLRs in CHB patients. This may mean that antiviral treatment together with TLR agonists could synergistically improve the effect of immune modulation in chronic HBV infection [41].

### 2.3. Modulation of TLR Signaling Pathway by HBV

Recent studies provided evidence that HBV can be recognized by the innate immune system as described above. However, compared with the hepatitis C virus (HCV), HBV induces negligible IFNs or ISGs in acute HBV-infected chimpanzees [10,42]. This may suggest that HBV has evolved strategies to avoid being recognized by the PRRs or to inhibit the PRR signaling pathways. The former theory was discussed above. However, several studies also support the latter hypothesis that the intracellular signaling pathways of TLRs could be interrupted by HBV components.

PDCs play a pivotal role in the innate immune response against viral infections by producing large amounts of type I IFNs through the TLR9 mediated signaling pathway. Xu et al. has demonstrated that HBsAg specifically suppressed TLR9-mediated IFN-α production by inhibiting expression and nuclear translocation of IRF7 [43]. Moreover, another study found that HBV particle internalization could inhibit TLR9-mediated production but not TLR7-mediated secretion of IFN-α by pDCs. Further analysis revealed that reduced expression of TLR9 was found in pDCs in the presence of HBV while HBV virions directly inhibited TLR9 promoter activity [23]. Wu et al. demonstrated that the supernatants from TLR3-activated LSECs suppressed HBV replication in hepatocytes [17]. However, a later study from the same group found that HBV components such as HBsAg, HBeAg, and HBV virions could evade TLR3-induced antiviral activity by suppressing the activation of IRF-3, NF-κB, and ERK1/2 in LSECs [44]. Our recent data revealed that HBsAg may play a crucial role in reducing the TLR3-mediated activation of LSECs by triggering IL-10 production from hepatic cells [45]. HBeAg was found to help reduce expression of TLR2 in CHB patients and impair induction of TNF-α in hepatic cell lines [21]. Later, studies from the same group demonstrated that HBeAg suppresses TIR-mediated activation of NF-κB and IFN-α promoters by disrupting homotypic TIR:TIR interaction [46]. Additionally, it could also inhibit IL-1β mediated NF-κB activation in hepatocytes [47]. The TLR3-mediated IFN-β induction in human hepatocytes was also suppressed by HBV polymerase, which interfered with IRF3 activation [48]. A recent study confirmed that HBV polymerase suppressed TNF-α, TLR3, and TLR4-induced NF-κB signaling by inhibiting the activity of IKKs. This was completed by interacting with Hsp90β in hepatoma cells [49].

Although these studies support the hypothesis that HBV may counter the host innate immune system by down regulating TLR expression and blocking the activation of downstream signaling pathways. Still, we need to pay more attention when considering these results in the context of natural HBV infection since a majority of experiments were performed either under the conditions of over-expressing a single viral protein or in cells that are not normally infected by HBV. Recently, using the dHepaRG cell model, Luangsay et al. demonstrated that physiologic HBV infection with HBV inoculum leads to a transient and abortive innate immune response compared with baculoviral vector-mediated transduction of HBV [14]. Detailed analysis revealed that this phenomenon may be attributed to the inhibition of TLR-3 and RIG-I/MDA5 signaling pathways by some factors presented in the HBV inoculum, but not HBsAg nor HBeAg [14]. The suppression of IFN-α was also found during acute (28 days after infection) or chronic (31 weeks after infection) HBV infection in vivo in the tupaia model [50]. Interestingly, during chronic infection, the suppression effect was found in all HBV-infected animals. However, during acute infection, the suppression was only found in HBV A2_JP4 infected but not HBV A2_JP1 infected tupaias [50]. This may suggest that HBV employs different mechanisms to resist the innate immune system in different periods of infection. Obviously, the interaction between HBV and innate immunity is more complicated in vivo. Thus, small animal models which support efficient HBV infection are needed to further investigate the field of HBV and TLR interaction.

## 3. Inhibition of HBV Replication by TLR-Mediated Innate Immune Responses

### 3.1. Cell Culture Models

Despite the fact that HBV infection induces negligible activation of innate immune responses in vivo, accumulated data provides evidence that activation of the TLR-mediated signaling pathway in hepatic cells and other immune cells could inhibit HBV replication in vitro and in vivo [16,17,18,19,20]. By using a model of murine hepatocytes replicating the HBV genome, Wu et al. showed that together with TLR3-activated murine LSECs, the TLR3- or TLR-4-activated KCs efficiently suppressed HBV replication in a soluble-molecule-dependent manner in vitro [17]. Detailed analysis demonstrated that the TLR3-mediated but not the TLR4-mediated antiviral effect is seen functioning through IFN-β. Other undefined cytokines might contribute to the antiviral effects of TLR4 in this model [17]. Using the dHepaRG cell model, Luangsay et al. demonstrated that strong antiviral activity against HBV was obtained in the presence of the ligands for TLR1/2, TLR4, and RIG-I/MDA-5 with the production of both cytokines IL-6 and IP-10 [26].

Several studies also provide evidence that TLR-mediated intracellular signaling pathways could also directly inhibited HBV replication in vitro. A study found that when HBV-replicating plasmids co-transfected with the TLR adaptors MyD88 and TRIF, or RLR adaptor IPS expression plasmids into hepatoma cells, the replication and antigen expression of HBV were suppressed [19], which was independent of the cytokines released from transfected hepatoma cells. These results suggested that the intracellular TLR or RLR signaling pathways were able to suppress HBV replication, and NF-κB activation was found to play the key role in adaptor-induced antiviral responses [19]. Our study also demonstrated that TLR2 activation resulted in the suppression of HBV or woodchuck hepatitis virus (WHV) in human hepatoma cells or primary woodchuck hepatocytes (PWHs). This antiviral effect was mainly dependent on adaptor molecules like TAK1, IRAK1/4, TRAF6 and molecules found in the downstream pathways like MAPK and PI-3k/Akt [18]. As such, TLR4 activation in PWHs by LPS led to a suppressed WHV replication in an IFN-independent manner [51]. Our recent study validated this hypothesis by demonstrating that overexpression transforming growth factor β-activated kinase 1 (TAK1), a key adaptor in TLR-mediated signaling pathway, efficiently inhibited HBV replication in vitro and in vivo [52].

### 3.2. In Vivo Models

Using HBV transgenic mice, McClary et al. found that TLR3 ligand poly(I:C) efficiently inhibited HBV replication by inducing type I IFNs [53]. Later, Isogawa et al. from the same group demonstrated that HBV replication in the liver could be abolished through intravenous injection with a single dose of ligands for TLR3, TLR4, TLR5, TLR7, or TLR9 in a type I IFN-dependent manner [16]. Recently, in an HBV-persistent mice model established by hydrodynamic injection of an HBV-genome-containing plasmid pAAV/HBV1.2 into the liver, Wu et al. reported that intravenous administration of poly(I:C) led to the clearance of HBV and the appearance of anti-HBs antibodies. Further analysis revealed that not only type I IFNs but also IFN-γ and CXCR-3 are essential for HBV clearance in this model, which indicates a coordinated action of innate and adaptive immune responses [54].

The WHV and its host, the eastern woodchuck, is a very valuable animal model system for the study of HBV infection [55]. Oral administration of TLR7 agonist GS-9620 in chronic WHV-infected woodchucks led to a sustained decrease of serum WHV DNA, hepatic replicative intermediates, and WHV cccDNA. This treatment also induced a loss of serum WHV surface antigen (WHsAg), obtained a sustained antibody response against WHsAg, and may further reduce the incidence of hepatocellular carcinoma in woodchucks [56]. Mechanistic analysis suggested that GS-9620 treatment induced a coordinated action of both intrahepatic CD8^+^ T cell, NK cell, B cell, and IFN response [56]. On the contrary, a TLR9 ligand CpG containing-oligonucleotides (ODNs) alone failed to suppress the WHV viremia despite its capacity of IFN and ISGs induction from the PBMCs in vitro and in vivo [57]. Recently, a RIG-I agonist SB 9200 has also shown a strong antiviral activity against WHV in a woodchuck model [58]. However, the antiviral effect of SB 9200 was momentary and associated with induction of IFN-α, IFN-β, and IFN-stimulated genes both in the blood and the liver [58].

The antiviral effect of TLR7 ligand GS-9620 were also verified in the HBV-infected chimpanzee model [20]. Four weeks oral administration of GS-9620 in HBV chronically infected chimpanzees led to long-term suppression of HBV DNA in the serum and the liver accompanied by a reduction of serum HBsAg and HBeAg levels [20]. The antiviral activity of GS-9620 in chimpanzees was associated with the production of IFN-α, the expression of ISGs, and the activation of natural killer cells as well as lymphocyte subsets [20]. Moreover, in a phase 1b trials of CHB patients, oral administration of GS-9620 induced a transient peripheral expression of ISG15 gene as well as the absence of detectable levels of serum IFN-α and related symptoms [59].

The above reports provide evidence that TLR-mediated direct or indirect antiviral activities could suppress HBV replication both in vitro and in vivo. However, the antiviral activities of TLR agonists were largely dependent on the induction of type I IFNs and were not as potent as those of the nucleos/tide analogues. Besides its direct antiviral effect, the cytokines induced by TLR activation could modulate the adaptive immune response against HBV, which are also important to terminate HBV infection. This will be discussed below.

## 4. Modulation of HBV-Specific Immune Responses by TLR Agonists

The host immune responses, especially the HBV-specific T and B cell responses, are key factors for determining the outcome of an acute HBV infection, including whether it progresses to recovery or becomes chronic [1,2]. An early, polyclone and multi-specific CD8^+^ T cell response against HBV proteins is always associated with viral clearance and the development of lifelong protection immunity in resolved people [3,4,5]. In contrast, a weak, transient and barely detectable HBV-specific CD8^+^ T cell response is correlated with viral persistence in chronically infected patients [6,7]. By using the WHV-infected woodchuck model, our recent studies showed that the therapeutic restoration of WHV-specific CD8^+^ T cell response (e.g., DNA vaccination or PD-1/PD-L1 blockage) led to viral clearance in chronically infected animals and development of antibody responses against WHsAg [60,61]. As such, the possibility of restoring an HBV-specific CD8^+^ T cell response in CHB patients represents an effective therapeutic strategy for terminating an HBV chronic infection.

In our recent study, using a HBV hydrodynamic injection mouse model, we found that the clearance of HBV was delayed in the mice lacking specific components of the IL-1R/TLR signaling pathway [25]. Further analysis revealed that the function of cytokine production but not cytotoxic activity of HBV-specific CD8^+^ T-cells was defective in the IL-1R/TLR signaling-deficient mice. This study suggested that the IL-1R/TLR signaling pathway might play an important role in removing the virus by mounting a functional HBV-specific CD8^+^ T-cell response [25]. Consistently, exogenous administration of TLR agonists have been shown to enhance HBV-specific immune responses and terminate the viral infection in several studies. The HBV-specific immune responses are impaired in the pAAV/HBV1.2 mice model and leads to persistence of the virus. TLR agonists were tested in this model to improve HBV-specific immune responses and break immune tolerance to the virus. Administration of a dual functional vector containing both an immunostimulating single-stranded RNA (ssRNA) and an HBx-silencing short hairpin RNA (shRNA) in these mice induced peripheral and hepatic HBV-specific CD8^+^ T cell responses and seroconversion of HBsAg, which led to removal of the virus. Further analysis revealed that the TLR7 signaling pathway and IFN-α/β receptor were required for inducing the systemic HBV-specific immune responses [62]. In another study, the same group found that the HBV genome contained unmethylated CpG ODNs (termed as HBV-CpG), which represented a TLR9 agonist and could stimulate robust IFN-α production of pDCs. Administration of nanoparticles containing HBV-CpG together with recombinant HBsAg (rHBsAg) immunization in mice enhanced immune response to HBsAg and skewed the CD4^+^ T helper cell response to the Th1 pathway. More importantly, this combined therapy strategy efficiently cleared the virus from HBV carrier mice and induced the anti-HBsAg response [63]. Recently, a study demonstrated that activation of the TLR9-mediated signaling pathway in the liver could induce intrahepatic aggregates of myeloid cells, which enable expansion of effector cytotoxic CD8^+^ T lymphocytes (CTLs) through OX40 stimulation. Importantly, in an HBV carrier mice model established by transferring the HBV genome via an adenovirus vector, HBV removal has been achieved by DNA vaccination and intravenous administration of the TLR9 ligand by expanding HBV-specific CTLs in the liver [64]. These studies indicate that TLR activation may break the immune tolerance state against HBV and enhance HBV-specific T or B cell responses, which might promote the clearance of the virus.

Recent evidence also demonstrated that TLRs may serve as costimulatory molecules on T cells [65]. Effector and memory CD4^+^ and CD8^+^ T cells express TLR2 on their surface and the activation of TLR2 by Pam3CSK4 could promote their proliferation, differentiation, and effector function in vitro and in vivo [66,67,68]. The costimulatory effect of TLR2 on CD8^+^ T cells was associated with increased T-bet transcription, which was dependent on the MyD88-Akt-mTOR- and protein kinase C-mediated signaling pathway [67]. In the tumor mice model, adoptive transfer of tumor antigen specific CD8^+^ T cells followed by Pam3CSK4 application consistently resulted in enhanced therapeutic efficacy [69,70]. The human CD8^+^ T cells were also found to express TLR3 and TLR9 on their surface and TLR stimulation directly promoted IFN-γ production in these cells [71,72,73]. Thus, these studies provided evidence that the TLR-mediated signaling pathway in T cells could directly promote T-cell proliferation and differentiation. However, further investigation for its use in treating chronic HBV infection is needed.

## 5. Potential Therapeutic Approaches for Eliminating Chronic HBV Infection Based on TLR Signaling Pathways

**Figure 1:**
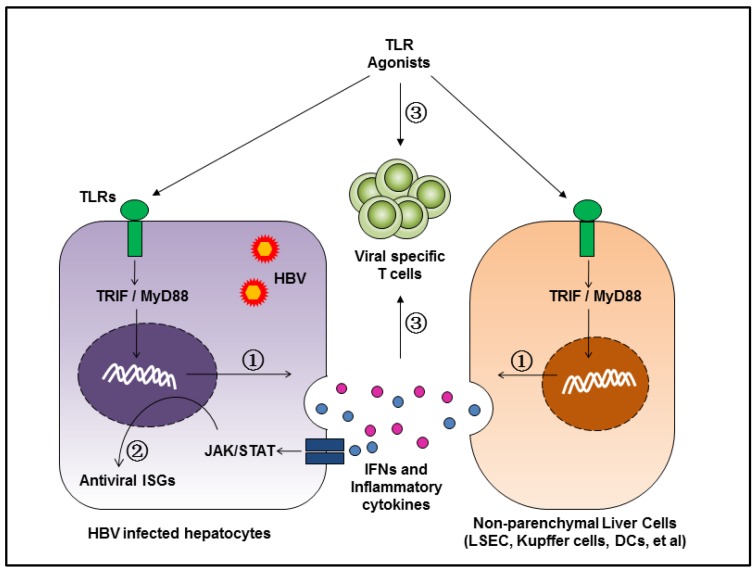
Hepatic activation of TLRs leads to suppression of HBV replication and restoration of HBV-specific adaptive immunity. TLRs are expressed in hepatocytes and hepatic non-parenchymal cells, including LSEC, Kupffer cells, DCs, and other cell types. Stimulation of TLRs by the respective ligands leads to the activation of downstream MyD88/TRIF-dependent signal pathways in hepatic cells and the production of pro-inflammatory cytokines, chemokines, and IFNs. The inhibition of HBV replication can be achieved by direct or indirect models: (1) the intracellular MAPK- and NF-κB-dependent signaling pathways trigger antiviral mechanisms; (2) IFNs and other yet unknown antiviral factors stimulate the expression of ISGs and other antiviral actions in hepatocytes; (3) the chemokines and inflammatory cytokines recruit HBV-specific T cells into the liver, promote T cell proliferation, and enhance the antiviral functions of HBV-specific CD8^+^ T cells. Therefore, TLRs inhibit HBV in the liver by activating both innate and adaptive responses.

Nowadays, two kinds of drugs are approved and recommended as referential therapies for CHB in clinical guidelines: PEG-IFN and nucleos/tide analogues. However, with these drugs, it is difficult to cure CHB, which requires achieving sustained and undetectable HBsAg and HBV DNA in serum with or without appearance of the anti-HBs antibody [74]. Thus, new therapeutic strategies are urgently needed, and there are already trials in preclinical or clinical phases. These novel antiviral therapies can be divided into two categories: (1) direct-acting antivirals (DAAs), which target specific steps in viral replication; and (2) host-targeting agents which suppress viral replication by modifying host cell functions [75]. Drugs targeting the TLR signaling pathway may represent the latter class. As discussed above, the activation of the TLR-mediated signaling pathway not only suppressed HBV replication in hepatocytes, but also led to restoration of HBV-specific immune responses (Figure 1).

Before the discovery of TLRs, a TLR3 ligand named complex of polyriboinosinic-polyribocytidylic acid (poly I-poly C) with poly-L-lysine and carboxymethylcellulose (polyICLC) was found to induce significant production of IFN in the serum of monkeys and chimpanzees [76]. Later, in chronic HBV-infected chimpanzees, the polyICLC showed effective antiviral activities against HBV through the induction of IFNs [77]. Another two kinds of TLR3 ligands, Ampligen and mismatched double-stranded RNA, were also recognized as IFN inducers and showed antiviral activities in hepatitis B virus (DHBV)-infected ducks [78,79]. As mentioned above, the TLR7 agonist GS-9620 also showed strong and sustained antiviral effects against WHV and HBV when it was administrated orally in the woodchuck [56] and chimpanzee models [20]. The antiviral activity of GS-9620 was mainly dependent on induction of IFNs from innate immune cells, such as monocytes, but not activation of the antiviral pathway in hepatocytes [80]. Moreover, the GS-9620 also showed the immune modulatory effect on an HBV-specific immune response by enhancing antigen presentation in hepatocytes [80]. In a phase 1b trial in CHB patients, oral administration of GS-9620 induced a transient peripheral expression of ISG15 with an undetectable level of serum IFN-α as well as related symptoms [59]. However, in this study, a single dose or two doses taken seven days apart of GS-9620 did not lead to a decrease of serum HBsAg or a decrease of HBV DNA levels. The reduced expression of TLR9 in PBMCs, pDCs, and liver-derived monocytes [23,36,37] may attribute to the low efficacy of GS-9620 in CHB patients. Recently, the safety and efficacy of GS-9620 has been assessed in CHB patients with suppressed HBV DNA levels by oral antiviral treatment. The efficacy of GS-9620 was assessed by reducing HBsAg after treatment. The results showed that no significant declines in HBsAg were observed in GS-9620 treated patients despite inducing ISG15 in PBMCs [81]. Thus, GS-9620 showed lower antiviral efficacy in CHB patients compared with that in chimpanzee models. Further studies are needed to improve the efficacy of GS-9620 in CHB patients. In a recent study, a panel of TLR agonists were used to stimulate mononuclear cells derived from human healthy and diseased liver tissues [82]. The results showed that the TLR8 ligand ssRNA40 could stimulate a high level of antiviral cytokine IFN-γ by activating innate immune cells in healthy and chronic HBV- or HCV-infected livers. The mucosal-associated invariant T cells and NK cells were identified as IFN-γ-producing cells after TLR8 activation. This study suggested a new therapeutic possibility of using TLR8 agonists for treating CHB [82]. Therefore, the agonists of TLR-3, -7 and -8 may serve as novel therapies for CHB, but further studies are needed to investigate their toxicity and tolerated range.

## 6. TLR Agonists as Adjuvants of HBV Vaccines

Even though there is still a long way to go before applying TLR agonists as therapeutic agents in treating CHB, TLR agonists are already used as adjuvants for prophylactic vaccines against HBV. Monophosphoryl lipid A (MPLA) is a lipid A derivative acquired from *Salmonella minnesota* R595 lipopolysaccharide (LPS) and used as a TLR4 based adjuvant. MPLA is considerably less toxic than LPS whilst maintaining better immunostimulatory activity [83]. Compared with conventional HBV vaccines, two improved HBV vaccines (Fendrix and Supervax) using MPLA adsorbed on either alum or oil as adjuvants [84], showed more ability to induce high levels of anti-HBs antibody in immune compromised patients [85] and healthy non-responder individuals [86]. Synthetic ODNs containing CpG motifs are constantly used as TLR9-based vaccine adjuvants and exert potent Th1-like immune enhancers by activating a TLR9-mediated signaling pathway [87]. Recently, two class B ODNs called 1018 ISS and CPG 7909 were used as adjuvants for developing new HBV vaccines. The mixture of CPG 7909 with rHBsAg absorbed to the alum showed good tolerance and enhanced vaccine immunogenicity. Further study suggested that this mixture not only induced a higher level of anti-HBs antibody, but also increased the pool of high-avidity antibodies by enhancing the late affinity maturation process [88,89]. Additionally, adding 1018 ISS to rHBsAg (Heplisav) also resulted in an enhanced immunogenicity and a higher level of anti-HBs antibodies compared with conventional vaccine Engerix-B [90,91,92,93]. More interestingly, the seroprotection rates were 100% versus 64% in rHBsAg plus 1018 ISS group compared to the rHBsAg only group [94]. Furthermore, such a vaccine formula may be more effective in an immune-compromised population such as populations with acquired immunodeficiency syndrome (AIDS) [95,96,97].

## 7. Conclusions

HBV affects 240 million people worldwide and represents a major global public health concern [98]. The antiviral treatment with PEG-IFN as well as nucleos/tide analogues can improve the prognosis of CHB patients by preventing the incidence of liver failure, cirrhosis, and hepatocellular carcinoma. However, it requires a life-long treatment in a great majority of the CHB patients and a functional cure in those patients is hard to achieve [74]. It is well accepted that the immune system, especially adaptive immunity, plays a key role in controlling HBV infection. Thus, restoring HBV-specific immune responses in CHB patients may be required for sustained viral control. So far, accumulated studies provide strong evidence that intrahepatic activation of TLR-mediated innate immune responses not only suppresses HBV replication in hepatocytes, but also enhances HBV-specific immune responses in the liver, which may lead to removing the virus. To achieve a functional cure for chronic HBV infection, a combined strategy with viral suppression by antiviral treatment, activation of TLR-mediated immunity, and restoration of HBV adaptive immunity may be needed. Further studies are required to explore this combination strategy in animal models and in clinical trials.
